# Sodium Malonate Inhibits the AcrAB-TolC Multidrug Efflux Pump of *Escherichia coli* and Increases Antibiotic Efficacy

**DOI:** 10.3390/pathogens11121409

**Published:** 2022-11-24

**Authors:** Allea Cauilan, Cristian Ruiz

**Affiliations:** Department of Biology, California State University Northridge, Northridge, CA 91330, USA

**Keywords:** malonate, sodium malonate, malonic acid, AcrAB-TolC, multidrug efflux pump, efflux pump inhibitor, EPI, *Escherichia coli*

## Abstract

There is an urgent need to find novel treatments for combating multidrug-resistant bacteria. Multidrug efflux pumps that expel antibiotics out of cells are major contributors to this problem. Therefore, using efflux pump inhibitors (EPIs) is a promising strategy to increase antibiotic efficacy. However, there are no EPIs currently approved for clinical use especially because of their toxicity. This study investigates sodium malonate, a natural, non-hazardous, small molecule, for its use as a novel EPI of AcrAB-TolC, the main multidrug efflux pump of the Enterobacteriaceae family. Using ethidium bromide accumulation experiments, we found that 25 mM sodium malonate inhibited efflux by the AcrAB-TolC and other MDR pumps of *Escherichia coli* to a similar degree than 50 μΜ phenylalanine-arginine-β-naphthylamide, a well-known EPI. Using minimum inhibitory concentration assays and molecular docking to study AcrB-ligand interactions, we found that sodium malonate increased the efficacy of ethidium bromide and the antibiotics minocycline, chloramphenicol, and ciprofloxacin, possibly via binding to multiple AcrB locations, including the AcrB proximal binding pocket. In conclusion, sodium malonate is a newly discovered EPI that increases antibiotic efficacy. Our findings support the development of malonic acid/sodium malonate and its derivatives as promising EPIs for augmenting antibiotic efficacy when treating multidrug-resistant bacterial infections.

## 1. Introduction

Resistance to antibiotics is a major threat worldwide because it makes previously curable infections hard to treat or untreatable, increases hospital costs, and negatively impacts medical procedures that rely on antibiotics, such as surgery, childbirth, and chemotherapy [1,2]. According to the Centers for Disease Control and Prevention, antibiotic-resistant bacteria infect nearly 3 million and directly kill nearly 36,000 people each year in the U.S. [2]. Between 2015 and 2050, antibiotic-resistant infections are projected to cause the premature deaths of 300 million people and USD 100 trillion in economic losses worldwide [1]. Finding new antibiotics is a lengthy and difficult process, which further exacerbates this problem [1,2,3]. Thus, novel approaches to counteract antibiotic resistance are desperately needed, especially to combat multidrug-resistant Gram-negative bacteria [1,2,3].

Among the major antibiotic resistance mechanisms, multidrug efflux (MDR) pumps are both one of the main challenges and most promising targets because they are present in all bacteria and significantly contribute to resistance to all antibiotic classes and to virulence [4,5,6,7]. MDR pumps contribute to intrinsic antibiotic resistance by expelling many structurally unrelated antibiotics and other toxic compounds [4,5,6,7], and function synergistically with the permeability barrier provided by the outer membrane of Gram-negative bacteria to prevent the accumulation of antibiotics [4,5,6,7,8]. For example, overexpression of AcrAB-TolC, which is the main MDR pump of *Escherichia coli* and other Enterobacteriaceae, confers resistance to nearly all classes of antibiotics, including last-resort antibiotics such as carbapenems, tigecycline and colistin [4,5,6,9,10,11]. Moreover, overexpression of the AcrAB-TolC pump facilitates the acquisition of mutations in antibiotic-target genes and of plasmids carrying genes for antibiotic-inactivating enzymes, leading to even higher levels of resistance [12,13,14,15].

Therefore, identifying efflux pump inhibitors (EPIs) capable of blocking MDR pumps is a very promising strategy for counteracting resistance to multiple antibiotics [4,5,6,16]. Prior studies have shown that EPIs of AcrAB-TolC and other MDR pumps successfully augment antibiotic efficacy [4,5,6,16,17,18,19,20,21,22]. However, none of the EPIs identified so far have been approved for therapy because of problems with their potency, bioavailability, pharmacokinetic properties, and their toxicity [5,16,23,24].

Drug repurposing represents a potentially faster and more cost-effective alternative to overcome these challenges [25,26,27]. This approach is gaining interest as a method for finding novel antimicrobials to treat multidrug-resistant infections because it focuses on drugs already approved or in advanced clinical trials [25,26,27]. Therefore, the safety, bioavailability, pharmacokinetics, pharmacodynamics, and dosing of these drugs are already known, which reduces costs and expedites their development [25,26,27]. A recent study in which drug repurposing was successfully applied to identify EPIs that potentiate the efficacy of fluoroquinolones against *Staphylococcus aureus* [28] further supports this approach for finding new EPIs. However, the application of drug repurposing for finding safe and effective EPIs remains an underexplored strategy, especially for EPIs that target Gram-negative bacteria.

Here, we investigated whether sodium malonate functions as an EPI and increases antibiotic efficacy in *E. coli*. Sodium malonate is a natural and broadly occurring metabolite known to inhibit the succinate dehydrogenase complex [29,30,31], and was previously studied as an osteoporosis therapeutic (as strontium malonate) in a human clinical trial [32]. Our findings show that sodium malonate is an EPI of the AcrAB-TolC MDR pump of *E. coli* and that it significantly increases the efficacy of ethidium bromide and the antibiotics minocycline, chloramphenicol, and ciprofloxacin. These findings indicate that malonic acid/sodium malonate, or their derivates, are promising antibiotic adjuvant candidates for combating multidrug-resistant Gram-negative bacteria.

## 2. Results and Discussion

### 2.1. Selection of Sodium Malonate as a Candidate Efflux Pump Inhibitor of the AcrAB-TolC Multidrug Efflux Pump of E. coli

We hypothesized that sodium malonate may function as an EPI of the AcrAB-TolC multidrug efflux pump of *E. coli* based on the following evidence. First, in a previous study using untargeted metabolomics [33], we found that strains deleted for the *acrB* or *tolC* genes showed increased intracellular levels of several intermediates of the tricarboxylic acid cycle, especially malic and fumaric acids. This finding suggests that these compounds, or their precursors or degradation products, may be substrates of the AcrAB-TolC pump [33]. Second, both malic and fumaric acids are dicarboxylic acids structurally related to malonic acid. This similarity, combined with the strong accumulation of dicarboxylic acids found in AcrAB-TolC-inactivated mutants, as discussed above, were the primary reasons for selecting sodium malonate for further studies. Moreover, our interest in studying malonic acid/sodium malonate also came from the fact that this compound is a well-known inhibitor of the succinate dehydrogenase and fumarate reductase enzymes [29,30], which are large, multi-subunit complexes located in the inner membrane [34], like the AcrB trimer component of the AcrAB-TolC pump [7,35].

Finally, sodium malonate was also selected because it is a generally safe compound (https://pubchem.ncbi.nlm.nih.gov/compound/8865, accessed on 1 April 2022). Sodium malonate/malonic acid is a naturally- and industrially-produced compound synthesized by many organisms; it is especially abundant in plants and as a fermentation product of some bacteria, and is also broadly present in living beings as malonyl-CoA, a key intermediate of fatty acid biosynthesis [31,36]. Moreover, malonate (as strontium malonate) has been studied as a therapeutic for osteoporosis in humans [32], and for its incorporation into bones in dogs [37,38], which further suggests that sodium malonate is safe and also has a favorable bioavailability profile.

### 2.2. Sodium Malonate Is an Efflux Pump Inhibitor of the AcrAB-TolC Multidrug Efflux Pump of E. coli Capable of Decreasing Efflux and Increasing the Antimicrobial Efficacy of Ethidium Bromide

**Figure 1:**
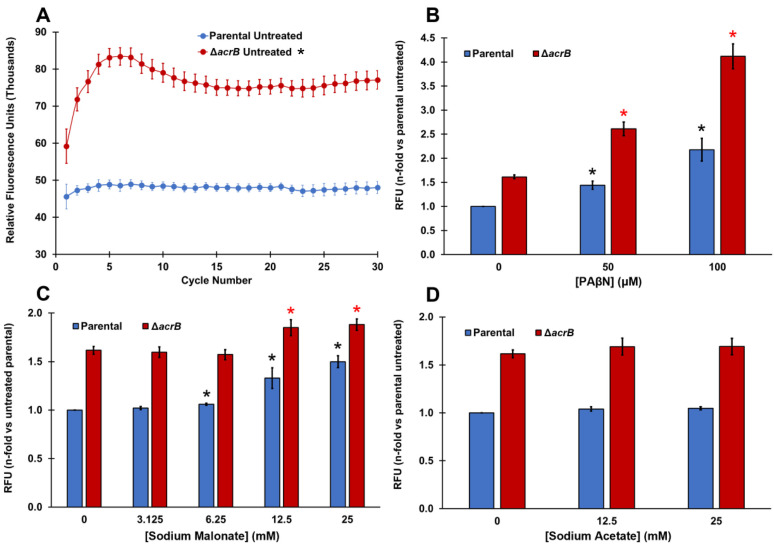
PAβN and sodium malonate, but not sodium acetate, are EPIs that prevent ethidium bromide efflux in *E. coli*. (**A**) Ethidium bromide accumulation was measured as the increase in relative fluorescence units (RFUs) for 30 cycles in the parental and Δ*acrB* strains. Results are presented as the average ± SEM (*n* = 6; i.e., 6 biological replicates, each including 3 technical replicates). RFU measurements remain stable between cycles 15 and 30. Ethidium bromide is a substrate of the AcrAB-TolC MDR pump and therefore it accumulated intracellularly significantly more in the Δ*acrB* strain than in the parental strain at all time points measured (*p* < 0.05; denoted with * next to the “Δ*acrB* untreated” legend at the top of the figure). (**B**–**D**) Ethidium bromide accumulation was measured as the increase in relative fluorescence units (RFUs) observed at cycle 30 for each strain in the presence of increasing concentrations of PAβN (**B**), sodium malonate (**C**), and sodium acetate (**D**). Results are presented as the average ± SEM (*n* = 3; i.e., 3 biological replicates, each including 3 technical replicates) and are shown as the n-fold change normalized to the untreated parental. Statistically significant differences (*p* < 0.05) between the untreated and treated are shown as * or *, for the parental and Δ*acrB* strains, respectively, for panels (**B**,**C**) (no differences for treatments were found in either strain for sodium acetate in panel (**D**)).

We used ethidium bromide (EtBr) accumulation assays to test whether sodium malonate acts as an EPI of the AcrAB-TolC MDR efflux pump of *E. coli* (Figure 1). EtBr is a well-known substrate of AcrAB-TolC [5,6,7] whose intracellular accumulation can be measured by the increase in fluorescence produced when EtBr binds to DNA [39]. For these assays, we tested a parental strain, as well as a Δ*acrB* strain because AcrB is the pump component that recognizes and binds to the substrates of AcrAB-TolC [4,5,6,7,35].

As expected, EtBr accumulated significantly more in the Δ*acrB* strain (60% increase in fluorescence) compared to the parental strain (Figure 1A). This difference between the parental and Δ*acrB* strains is similar to that reported by Coldham et al. [39] in *Salmonella*. We next tested phenylalanine-arginine-β-naphthylamide (PAβN), which is a well-known EPI of AcrAB-TolC and other MDR pumps [5,39,40]. The addition of PAβN produced a dose-dependent increase in the intracellular levels of EtBr in both the parental and Δ*acrB* strains (Figure 1B). A dose-depended increase in EtBr accumulation caused by PAβN in the parental strain is consistent with the dose-dependent increase for this EPI previously found in *Salmonella* using Hoechst 33342, a dye and AcrAB-TolC substrate that functions in a similar manner to EtBr [39]. To our knowledge, this is the first report of a dose-depended effect in EtBr accumulation caused by PAβN in an *E. coli* Δ*acrB* mutant.

Interestingly, sodium malonate also caused a significant and dose-dependent increase in intracellular EtBr at concentrations of 6.25 mM and higher, mainly in the parental strain (Figure 1C). Of note, we observed a 50% increase in EtBr accumulation (fluorescence) when we tested 25 mM sodium malonate in the parental strain (Figure 1C), which is similar to the increase observed when the AcrAB-TolC pump was genetically inactivated, i.e., in the Δ*acrB* strain (Figure 1A). On the contrary, when tested in the Δ*acrB* strain, sodium malonate only had a small effect, with the maximum increase in fluorescence observed being 16% at 25 mM (Figure 1C). Overall, these findings indicate that sodium malonate is a newly discovered EPI, and although it may also inhibit other MDR pumps in *E. coli*, most of the prevention of EtBr efflux by sodium malonate occurs via the inhibition of the AcrAB-TolC pump.

To investigate whether such an effect by sodium malonate was specific, we also tested another small organic acid salt, sodium acetate, and found very minimal changes in EtBr accumulation (less than 5% increase in fluorescence) for both the parental and Δ*acrB* strains at all concentrations tested (Figure 1D).

**Figure 2:**
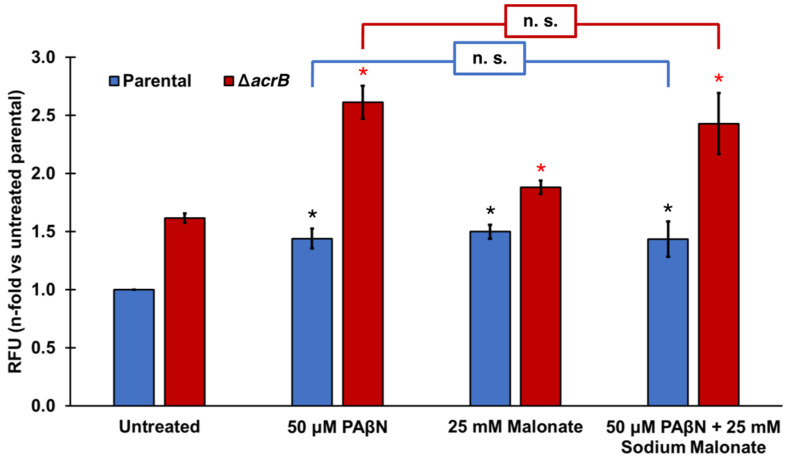
Effect of the EPIs PAβN and sodium malonate individually and in combination on ethidium bromide efflux. Ethidium bromide accumulation was measured in *E. coli* parental and Δ*acrB* strains as the increase in relative fluorescence units (RFUs) observed at cycle 30 for each strain and treatment. Results are presented as the average ± SEM (*n* = 3; i.e., 3 biological replicates, each including 3 technical replicates) and are shown as the n-fold change normalized to the untreated parental. Statistically significant differences (*p* < 0.05) between the untreated and treated are shown as * or *, for the parental and Δ*acrB* strains, respectively. Sodium malonate did not significantly change the effect of PAβN in either strain (noted as n.s.).

Finally, we tested the effect of adding PAβN and sodium malonate simultaneously in the ethidium bromide accumulation assay (Figure 2). At the concentrations tested (50 μM PAβΝ and 25 mM sodium malonate), both EPIs did not seem to function in an antagonistic, additive, or synergistic manner (Figure 2). Of note, Figure 1B shows that significantly greater EtBr accumulation occurred at 50 μM PAβΝ when the *acrB* gene was deleted, or at 100 μM PAβΝ compared to 50 μM PAβΝ in both the parental and Δ*acrB* strains. These findings suggest that the lack of effect observed when sodium malonate was added to assays with 50 μM PAβΝ (Figure 2) was not the result of the assay being saturated.

**Figure 3:**
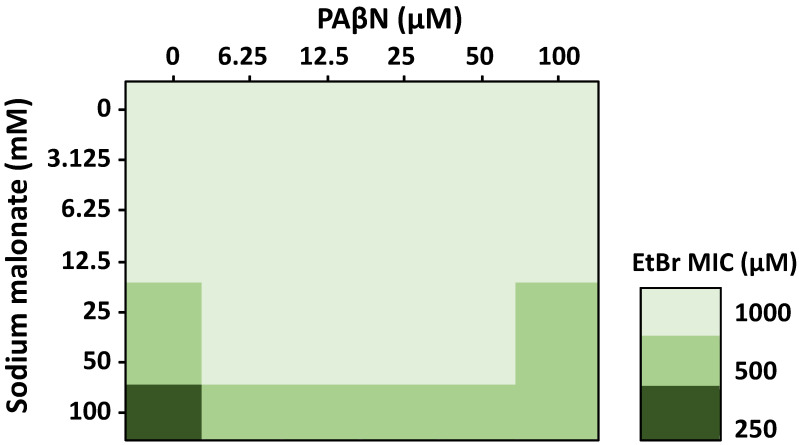
Effect of the EPIs sodium malonate and PAβN in combination on the antimicrobial activity of EtBr measured by 3D-checkerboard assays. Sodium malonate increased the efficacy of EtBr at concentrations of 25 to 100 mM (*n* = 3 biological replicates, each with one technical replicate), and all changes were statistically significant (*p* < 0.05). PAβN at concentrations of 6.25 to 50 μM decreased by 2-fold the MIC of EtBr at concentrations of sodium malonate of 25–50 mM; PAβN at 100 μM only decreased the efficacy of the EtBr-100 mM sodium malonate combination. Data are presented as the average of 3 biological replicates, each including 1 technical replicate. All differences were statistically significant (*p <* 0.05).

To further investigate the effects of sodium malonate and PAβΝ in combination, we performed 3D checkerboard assays with both EPIs and EtBr, using the parental strain (Figure 3). Whereas EtBr was tested at concentrations up to its MIC, sodium malonate and PAβΝ were tested at concentrations well below their MIC, which was 1 M for sodium malonate (Table 1), and 932 μM (512 μg/mL) for PAβΝ [41], to focus on their EPI effects, and not the antimicrobial effects these EPIs may have at greater concentrations. Therefore, formal FICI scores could not be calculated, but rather the assay showed the EPI effect of sodium malonate and PAβΝ on potentiating the antimicrobial effects of EtBr.

In the absence of PAβΝ, we found that sodium malonate had no effect on the MIC of EtBr at sodium malonate concentrations of 12.5 mM and lower, but decreased the MIC of EtBr by two-fold at 25-50 mM (Figure 3). When sodium malonate was tested at 100 mM, it decreased the MIC of EtBr by four-fold, from 1000 μM to 250 μM. These findings are consistent with our accumulation assay results showing that sodium malonate at 25 mM was an EPI that significantly prevented EtBr efflux via the AcrAB-TolC pump (Figure 1C).

In the absence of sodium malonate, PAβΝ at concentrations up to 100 μM did not change the MIC of EtBr (Figure 3), despite the increased EtBr accumulation found for this compound, especially when tested at 100 μM (Figure 1B). This finding was unexpected. However, we speculate that it might be related to PAβΝ producing other effects, such as inducing the expression of AcrAB-TolC or other MDR efflux pumps capable of effluxing EtBr, which might contribute to negating the AcrAB-TolC-inhibiting effects of PAβΝ. In fact, while the EtBr accumulation assays occur during a short time frame, MIC assays have 18-h incubations, which would allow for changes in gene expression. Moreover, we have observed before that 100 μM of PAβΝ, which is a concentration at which we expected to detect a change in the MIC of EtBr, induced the expression of *acrAB*. Finally, it is possible that changes in the MIC of EtBr might have been observed using higher PAβΝ concentrations. However, such concentrations were not tested because of the well-known effects of PAβΝ on increasing inner and outer membrane permeability at high concentrations [42], which would have confounding effects when studying its efflux-related effects.

Interestingly, when tested in combination, we found that 6.25 to 50 μM PAβN concentrations decreased by two-fold (i.e., the MICs of EtBr increased by two-fold) the efficacy of 25–100 mM sodium malonate concentrations in potentiating the antimicrobial effects of EtBr. At 100 μM PAβN, we found a similar two-fold decrease in the EtBr potentiating effects of sodium malonate, but only for the 100 mM sodium malonate-EtBr combinations (Figure 3). As discussed above, FICI scores could not be obtained because sodium malonate and PAβN were tested at below MIC concentrations to focus on their EPI activity and not their antimicrobial effects. However, our overall findings show a moderate antagonism by PAβN on the efficacy of sodium malonate in decreasing the MIC of EtBr. Further experiments will be necessary to fully characterize this phenomenon.

### 2.3. Sodium Malonate Increases the Efficacy of Ethidium Bromide and Antibiotics in E. coli

We next used minimum inhibitory concentration (MIC) assays to study the growth inhibitory effect of sodium malonate, and whether its EPI activity increased the efficacy of not only EtBr, but also of three different antibiotics known to be substrates of AcrAB-TolC [5,6,7], minocycline, chloramphenicol, and ciprofloxacin (Table 1). Both the parental and Δ*acrB* strains were tested to study the role of inhibition of the AcrAB-TolC pump in any antimicrobial-potentiating effects found for sodium malonate. The effect of 50 μM PAβΝ was also studied for comparison. We observed that sodium malonate had a very small growth inhibitory effect on its own (MIC of 1 M for both the parental and Δ*acrB* strains; Table 1).

When we tested EtBr in combination with 100 mM sodium malonate, the MIC of EtBr decreased by four-fold in the parental strain, but only by two-fold in the Δ*acrB* strain (Table 1). These findings are consistent with our accumulation assay results showing that sodium malonate is an EPI that prevents EtBr efflux by inhibiting the AcrAB-TolC pump, and also independently from its effect on AcrAB-TolC (Figure 1C). Further studies are in progress to identify the other MDR pump(s) inhibited by sodium malonate and to determine whether such inhibition is only relevant in the absence of a functional AcrAB-TolC pump.

Interestingly, adding sodium malonate (or PAβΝ) decreased the MIC of minocycline in the parental strain by four-fold, whereas it had no effect in the Δ*acrB* strain (Table 1). These results indicate that the increase in minocycline efficacy produced by sodium malonate or PAβΝ is fully dependent on the AcrAB-TolC pump, and further supports the role of these small molecules as EPIs of this pump.

Of note, we also found that sodium malonate decreased by two-fold the MICs of chloramphenicol and ciprofloxacin, although such an effect occurred both in the parental and Δ*acrB* strains for chloramphenicol and only in the Δ*acrB* strain for ciprofloxacin (Table 1). PAβΝ also decreased the MIC of chloramphenicol, but only in the parental strain and to a higher degree (four-fold; Table 1). As with sodium malonate, PAβΝ only decreased the MIC of ciprofloxacin in the Δ*acrB* strain, although the effect was greater (four-fold; Table 1). Potentiation of the antimicrobial effect of ciprofloxacin in *E. coli* when adding PAβΝ (by two-fold in the parental and by >two-fold in the Δ*acrB* strain) has been reported before [41], although using PAβΝ concentrations about twice the concentration tested here.

The results with chloramphenicol and ciprofloxacin indicate that sodium malonate can increase the efficacy of some antibiotics independently or even in the absence of the AcrAB-TolC pump. As in the case of EtBr, we hypothesize that such an effect may occur by this EPI inhibiting other MDR efflux pumps of *E. coli*, which would be consistent with the AcrAB-TolC-dependent and -independent inhibitory effects observed for sodium malonate in our EtBr accumulation assays (Figure 1C). Thus, we hypothesize that inhibition of other MDR pumps would explain why sodium malonate increased the efficacy of chloramphenicol in both the parental and Δ*acrB* strains. For ciprofloxacin, because sodium malonate only increased its efficacy in the Δ*acrB* strain, we hypothesize that sodium malonate inhibited other MDR pump(s) whose effect was only apparent in the absence of the AcrAB-TolC pump. In fact, it is well-known that AcrAB-TolC is the main MDR pump of *E. coli* and that the role of other MDR pumps in antibiotic efflux is often only apparent in the absence of AcrAB-TolC [5,6,7,43,44]. However, other efflux-independent effects of sodium malonate on the uptake, targets, or activity of ciprofloxacin cannot be discarded.

Finally, it is important to note that, although sodium malonate functioned as an effective EPI to increase the efficacy of several antibiotics against *E. coli*, further studies are necessary to study its effectiveness for other antibiotics and bacteria. For example, one limitation of sodium malonate would be its use against some pathogens such as *Klebsiella pneumoniae* [45] known to degrade malonate via malonate decarboxylases. Nevertheless, this study provides a foundation for further developing malonate/malonic acid derivatives with increased EPI activity, and potentially more resistant to degradation by malonate decarboxylases, for treating different Gram-negative bacteria.

### 2.4. Molecular Docking Suggests That Sodium Malonate Possibly Inhibits the AcrAB-TolC Pump by Binding to the Proximal Binding Pocket and other Locations in the Porter Domain of AcrB

**Figure 4:**
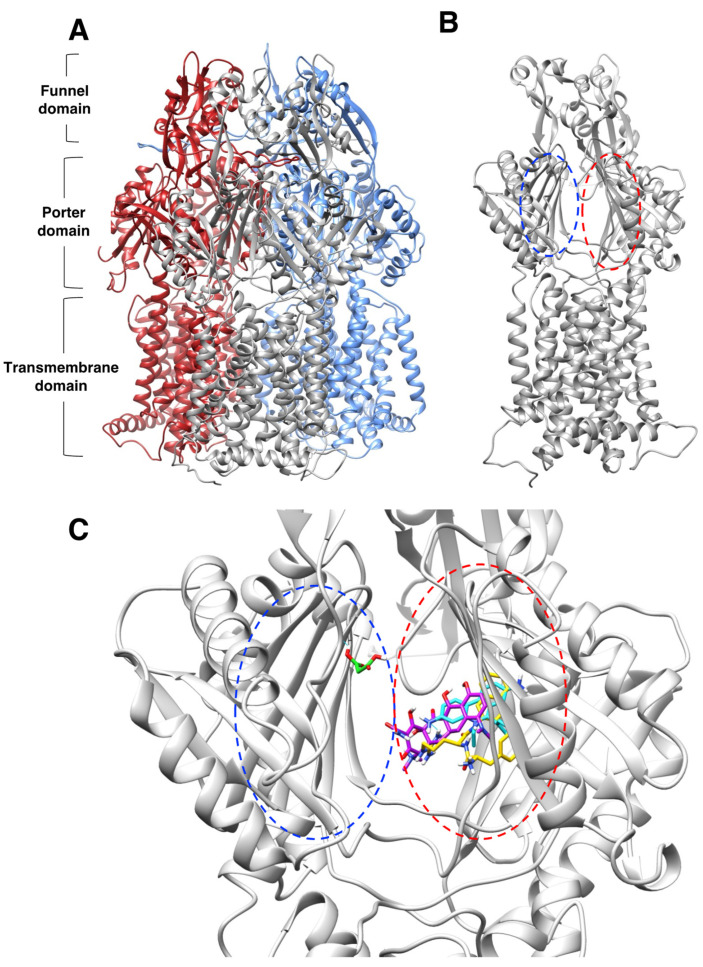
Molecular docking characterization of ethidium bromide, minocycline, PAβN, and sodium malonate binding to AcrB. (**A**) Overall structure of the AcrB homotrimer. (**B**) AcrB monomer, regions containing the proximal and distal binding pockets according to ref. [41] are indicated as blue and red dotted ovals, respectively. (**C**) Zoom view of the proximal (blue dotted oval) and distal binding pockets (red dotted oval) of AcrB showing molecular docking results for the four ligands tested. Ligands are colored by atom, and carbon atoms are colored in cyan (ethidium bromide), purple (minocycline), PAβN (yellow), and green (sodium malonate). The scores for the docking result shown for each ligand were: EtBr: −9.6; minocycline: −8.2; PAβN: −10.2 (only one of two similar poses with the same score is shown); and sodium malonate: −4.3. The poses shown represent the highest score poses found to bind to the distal or proximal binding pockets. Full docking results for all ligands are provided in Figure 5.

**Figure 5:**
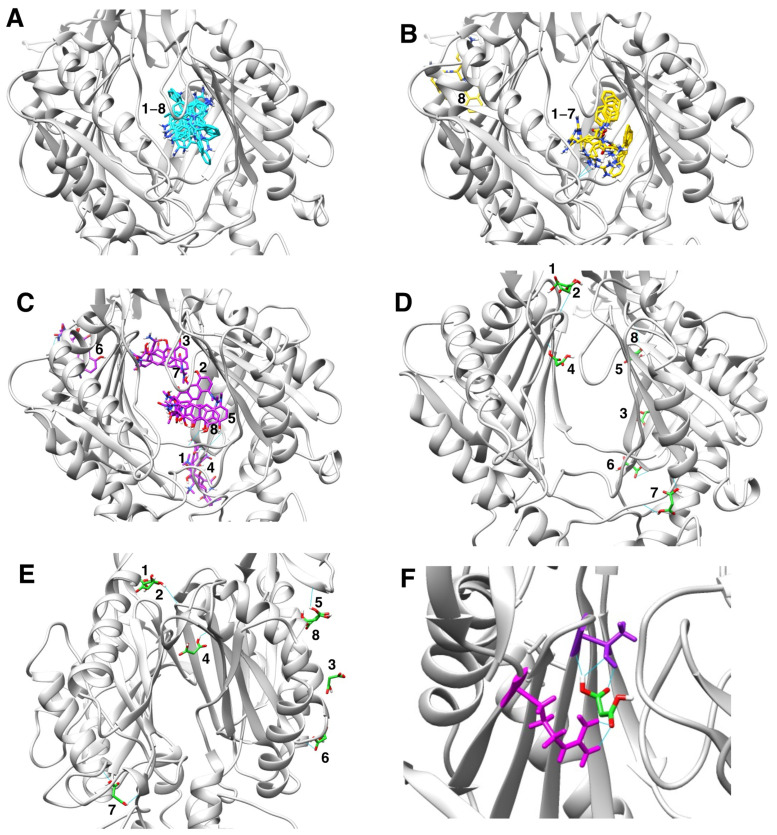
Molecular docking characterization of ethidium bromide, minocycline, PAβN, and sodium malonate binding to AcrB, showing all top 8 results obtained from AutoDock Vina. (**A**) Ethidium bromide (cyan): all 8 poses bound very closely in the distal binding pocket with scores ranging from -9.6 (top score; also shown in Figure 4C) to −8.4. (**B**) PAβN (yellow): 7 poses bound very closely in the distal binding pocket with scores ranging from -10.2 (top score; also shown in Figure 4C) to −9.4. Only one pose (no. 8; score = −9.2) was found outside the distal binding pocket. (**C**) Minocycline (purple): poses were found both in the distal binding pocket (pose no. 2, score −8.2, also shown in Figure 4C; pose no. 5, score = −8.1; and pose no. 8, score = −7.8) and in other areas of the AcrB porter domain (pose no. 1, score = −8.4; pose no. 3, score = −8.2; pose no. 4, score = −8.1; pose no. 6, score = −8.0; and pose no. 7, score = −7.8. (**D**,**E**) Sodium malonate (green) poses are shown in two different AcrB orientations. No poses were found in the distal binding pocket, and only one pose (no. 4, score = −4.3; also shown in Figure 4C) was found in the proximal binding pocket. The other poses were found in different areas of the porter domain (poses no. 1, no. 2, no. 3, no. 5, no. 6, no. 7, and no. 8, which had scores of −4.5, −4.5, −4.4, −4.2, −4.2, −4.4 and −4.2, respectively). (**F**) Zoom view of sodium malonate (green) bound to the proximal binding pocket (pose no. 4), which was stabilized with three hydrogen bonds (in cyan) with threonine-87 (shown in purple above sodium malonate) and two hydrogen bonds with arginine-815 (shown in magenta).

We next used molecular docking to further investigate the mechanism by which sodium malonate might prevent the efflux of EtBr and minocycline, the two antimicrobials for which sodium malonate inhibition was mostly (EtBr) or completely (minocycline) AcrAB-TolC-dependent (Table 1). We found that EtBr and minocycline, as well as PAβN, generally bound with most of their poses and their highest scores (−9.6 for EtBr, −8.2 for minocycline, and −10.2 for PAβN) to the distal binding pocket of AcrB, and that such binding was partially overlapping (Figure 4 and Figure 5A–C). The only exception was minocycline, for which although several binding results were in the distal binding pocket, the highest score (−8.4) was found outside this pocket (Figure 5C). These findings, in particular their binding to the distal binding pocket shown in Figure 4C, are in general agreement with prior results obtained for these molecules using crystallography, mutagenesis, docking, and/or molecular dynamics simulations [41,46,47,48,49]. In contrast, we found that sodium malonate did not bind to the distal pocket but bound to the proximal binding pocket (score = −4.3) and other regions of the porter domain with similar scores (−4.5 to −4.2) (Figure 4C and Figure 5D,E). Given that sodium malonate is a much smaller molecule than the other ligands tested, and thus has a smaller binding surface, it is not surprising that the overall binding scores found for this ligand were lower. Of all results for sodium malonate (Figure 5D,E), only pose no. 6 overlapped with the other ligands (minocycline poses no. 1 and no. 4), whereas poses no. 5 and no. 8 were found to be nearby minocycline pose no. 7. However, these three minocycline poses were outside of the ligand binding pockets, which questions their biological relevance for minocycline transport via AcrB.

We hypothesize that the EPI effects found for sodium malonate are dependent on its binding to the distal pocket, mediated by interactions with threonine-87 and arginine-815 (Figure 5F). However, we cannot discard that binding of sodium malonate to any of the other identified locations in the porter domain of AcrB (Figure 5D,E) also contribute, or even are completely responsible, for the EPI effects of this molecule. Another possibility is that the inhibition of the AcrAB-TolC pump found for sodium malonate is the result of additive effects caused by the simultaneous binding of this compound to the proximal binding pocket and/or several other locations identified in the porter domain. Future experiments using molecular dynamics simulations to study the stability of the poses identified for this compound, and mutagenesis experiments to test the key AcrB residues predicted to interact with sodium malonate will be necessary to confirm the binding site(s) of sodium malonate in AcrB, and to fully characterize its mechanism of action.

The export of drugs by AcrB appears to occur by cooperative rotation between three different monomer conformations (loose, tight, and open). Drugs access AcrB via the proximal binding pocket in the loose conformation, which is followed by a conformational change to the tight state that moves drugs to the distal binding pocket, and a second conformational change to the open state, in which drugs are then transported through the exit channel [5,7,35,50]. Prior studies have indicated that PAβN and other EPIs can inhibit drug efflux by directly hindering drug binding, or by binding to a different location than the drugs and causing a conformational change in AcrB that either prevents drug binding or restricts the dynamics of drug export of AcrB [41,46,47,48,49]. Although our preliminary studies suggest that sodium malonate might function by the later mechanism, future studies will be necessary to fully characterize the mechanism of action of this novel EPI.

## 3. Materials and Methods

### 3.1. Bacterial Strains and Culture Conditions

The bacterial strains used in this study are *Escherichia coli* BW25113 (parental strain [51]; F^–^ λ^–^ Δ*(araD–araB)567* Δ*lacZ4787*(::*rrnB-3*) *rph-1* Δ*(rhaD–rhaB)568 hsdR514*), and *E. coli* CR5000 (BW25113 Δ*acrB* [52]). Both strains were routinely grown in lysogeny broth (LB; 10 g/L tryptone, 5 g/L yeast extract, 10 g/L NaCl; Fisher Scientific, Hampton, NH, USA) at 37 °C with 200 rpm agitation.

### 3.2. Ethidium Bromide Accumulation Assays

Ethidium bromide accumulation assays to study efflux pump inhibitors (EPIs) were performed as previously described [39], with the following modifications. Briefly, cultures of *E. coli* strains BW25113 or CR5000 were grown in LB at 37 °C overnight, and then subcultured 1:1000. Cells then were grown for 2.5–3 h at 37 °C to mid-exponential phase before pelleting them by centrifugation at 10,000× *g* for 3 min. Next, cell pellets were resuspended in 1× Phosphate Buffered Saline (PBS; 137 mM NaCl, 2.7 mM KCl, 10 mM Na_2_HPO_4_, 1.8 mM KH_2_PO_4_, pH 7.4) to an OD_600nm_ of 0.1 measured with a WPA CO8000 cell density meter (Biochrom US, Holliston, MA, USA). Ethidium bromide accumulation reactions (200 µL each) were then prepared in black clear-bottom 96-well microplates by adding 160 µL of resuspended cells, 20 µL of ethidium bromide in PBS (Fisher Scientific, Waltham, MA, USA; 2.5 µM final concentration), and 20 µL of PBS (untreated) or EPI/compound prepared in PBS. The compounds tested were phenylalanine-arginine-β-naphthylamide (PAβN; positive control; Fisher Scientific, Waltham, MA, USA), sodium malonate (malonic acid disodium salt hydrate; Thermo Fisher Scientific, Waltham, MA, USA) and sodium acetate (negative control; Fisher Scientific, Waltham, MA, USA). Wells with PBS and compounds but not with cells were used as blanks to subtract the fluorescence caused by the tested compounds. The relative fluorescence units (RFUs) of each well were then measured at 37 °C using a Perkin Elmer (Waltham, MA, USA) Victor Nivo 5S multimode plate reader. RFUs were measured five times for each cycle, over the course of 30 cycles (75 min). The excitation and emission filters used were 515/30 nm and 600/10 nm respectively. Each strain and treatment was tested using three to six biological replicates (see Figure 1 legend for full details), each having three technical replicates.

### 3.3. Minimum Inhibitory Concentration (MIC) Assays

The minimal inhibitory concentrations (MICs) of sodium malonate and the antimicrobials ethidium bromide, minocycline, chloramphenicol, and ciprofloxacin for the parental (BW25113) and Δ*acrB* (CR5000) *E. coli* strains were determined by using a previously described standard broth microdilution method [53], with the following modifications. Briefly, cells were first grown overnight in LB at 37 °C with agitation, then diluted in Mueller Hinton broth (MHB; Fisher Scientific) to a density comparable to a 0.5 MacFarland, and then further diluted 1:100 in MHB. Then, MIC assays were conducted in 96-well microplates using 150 µL cultures containing 50 µL of cells in MHB, and 100 µL of two-fold serial dilutions of sodium malonate (to determine the MIC of this compound) or the tested antimicrobials prepared in MHB with or without 100 mM sodium malonate (or 50 μM PAβΝ) (to determine the effect of sodium malonate or PAβΝ on the MIC of the antimicrobials tested). Finally, the OD_600nm_ of each well was measured after 18 h of growth at 37 °C using a VICTOR Nivo S5 multimode plate reader, and the MIC was determined as the compound concentration that produced a 95% reduction or greater in growth compared to the untreated. MIC assays for each strain and treatment were performed using three biological replicates, each having three technical replicates.

Checkerboard 3D assays to study the individual and combined EtBr-potentiating effects of sodium malonate and PAβΝ were performed essentially as previously described [54] except for testing sodium malonate and PAβΝ at concentrations in which they act as EPIs and not as antimicrobials. Briefly, the MIC for EtBr at each sodium malonate and/or PAβΝ tested (see Figure 3) was determined as described above, using two-fold EtBr dilutions series that also contained sodium malonate and/or PAβΝ at increasing concentrations. Assays were performed using three biological and one technical replicate for each EtBr-sodium malonate-PAβΝ concentration tested.

### 3.4. Statistical Analysis

Statistically significant differences in ethidium bromide accumulation and MIC assays were determined by *t*-test (two independent samples with equal variance, two-tailed distribution) performed using Microsoft^®^ Excel 2019 v16.67 software. The averages, standard error of the mean (SEM; for clarity, the SEM is only shown for ethidium bromide accumulation assays, i.e., Figure 1 and Figure 2), and statistical significance (*p* < 0.05) are provided in Figure 1, Figure 2 and Figure 3 and their legends, as well as in Table 1.

### 3.5. Molecular Docking

Molecular docking was performed using UCSF Chimera v1.16 and AutoDock Vina v1.1.2 [55] software as previously described [56]. Briefly, the structure of AcrB (PDB:2GIF; trimeric structure in which each monomer is in one of the three consecutive states of the transport cycle [50]) was downloaded as a .pdb file from the RCSB Protein Data Bank (https://www.rcsb.org/ accessed on 1 July 2022), and ligands were downloaded as 3D conformers .sdf files from PubChem (https://pubchem.ncbi.nlm.nih.gov/ accessed on 1 July 2022; EtBr CID 3624, minocycline CID 54675783, PAβN CID 443301, sodium malonate CID 867). Both AcrB and ligands were prepared in Chimera by adding hydrogens, removing water molecules, and adding Gastgeiger charges, and then saved as .mol2 files. Finally, blind docking between nearly all of the AcrB trimer and the ligands studied was performed using AutoDock Vina in Chimera, by using a grid of 100 × 105 × 100 Å, an exhaustiveness value of 8, and the default AutoDock Vina options. Because the four ligands bound overall in the porter domain of all three monomers, with their highest score in the ligand binding cavity of the AcrB B monomer, docking with AutoDock Vina was repeated using a second refined grid of 55 × 55 × 50 Å that included nearly all of the porter domain of this monomer, centering the grid around its ligand binding region.

## 4. Conclusions

There is a desperate need to identify novel therapeutical agents to treat multidrug-resistant Gram-negative bacteria. Targeting their multidrug efflux (MDR) pumps is a very promising strategy because they are major contributors to intrinsic and acquired antibiotic resistance. However, there are currently no efflux pump inhibitors (EPIs) approved for use in humans because of their toxicity and other challenges. This study identifies sodium malonate as a novel EPI of the AcrAB-TolC and potentially other MDR pumps of *E. coli*. We show that this small molecule successfully increases the efficacy of ethidium bromide and the antibiotics minocycline, chloramphenicol, and ciprofloxacin. Moreover, our results suggest that sodium malonate might function by binding to multiple locations in the porter domain of AcrB, including the proximal binding pocket. Overall, these findings are significant for several reasons. First, they provide the proof of principle that small molecules structurally related to cellular metabolites previously found to accumulate in AcrAB-TolC mutants can act as EPIs of this important MDR pump. Second, these results show for the first time that drug repurposing can successfully be used to find novel and potentially safer EPIs of Gram-negative MDR pumps. Third, our findings provide a rationale for further studying the EPI activity, antibiotic augmenting effects, stability, and structure-activity–relationships of malonic acid/sodium malonate, and their derivatives, in a broader context involving additional antibiotics, efflux pumps, and pathogens. Ultimately, this study provides a foundation for further developing sodium malonate derivatives as safe and effective EPIs for improving the efficacy of antibiotics used to treat multidrug-resistant infections caused by Gram-negative bacteria.

## Figures and Tables

**Table 1:** Minimum inhibitory concentration of sodium malonate and various antimicrobials in the absence or presence of 100 mM sodium malonate or 50 μM PAβN for the *E. coli* parental and ∆*acrB* strains. Data are presented as the average of 3 biological replicates, each including 3 technical replicates. All differences were statistically significant (*p <* 0.05).

Compound	MIC (Parental Strain)			MIC (Δ*acrB* Strain)		
	− EPI	+ Sodium Malonate	+ PAβN	− EPI	+ Sodium Malonate	+ PAβN
Sodium malonate	N/A	1 M	N/A	N/A	1 M	N/A
Ethidium bromide	1000 μM	250 μM	1000 μM	31.25 μM	15.6 μM	15.6 μM
Minocycline	0.5 μg/mL	0.125 μg/mL	0.125 μg/mL	0.0625 μg/mL	0.0625 μg/mL	0.0625 μg/mL
Chloramphenicol	5.0 μg/mL	2.5 μg/mL	1.25 μg/mL	1.25 μg/mL	0.625 μg/mL	1.25 μg/mL
Ciprofloxacin	0.02 μg/mL	0.02 μg/mL	0.02 μg/mL	0.005 μg/mL	0.0025 μg/mL	0.00125 μg/mL

## Data Availability

All data are included in the manuscript.
